# Granulosa-cell tumor after ovarian stimulation: A case report

**Published:** 2018-06

**Authors:** Zohreh Yousefi, Hekmat Khalilifar, Amir Hosein Jafarian, Behrouz Davachi, Leila Mousavi Seresh, Nooshin Babapour, Laya Shirinzadeh, Mina Baradaran

**Affiliations:** 1 *Department of Obstetrics and Gynecology, Faculty of Medicine, Mashhad University of Medical Sciences, Mashhad, Iran. *; 2 *Kosar IVF Center, Mashhad, Iran.*; 3 *Department of Pathology, Faculty of Medicine, Mashhad University of Medical Sciences, Mashhad, Iran.*; 4 *Department of Radiology, Faculty of Medicine, Mashhad University of Medical Sciences, Mashhad, Iran.*

**Keywords:** Ovarian stimulation, Granulosa-cell tumor, Ovarian cancer

## Abstract

**Background:**

Ovarian superovulation and increased follicle-stimulating hormone concentration for infertility treatment may be the risk factors of developed granulosa-cell tumor. The aim of this report is to introduce a case of granulosa-cell tumor which was discovered after ovarian stimulation.

**Case:**

A 31-yr-old woman with clinical presentation of massive abdominal distention was referred to the gynecology and oncology department of an academic hospital, Mashhad University of Medical Sciences in Aug 2017. She had the history of secondary infertility and was undergoing In Vitro Fertilization protocol and ovarian stimulation, but, the cycle was canceled. The patient suffered from gradual abdominal distention one month after the end of IVF procedure despite pregnancy failure. 2-3 months after management of the ovarian hyperstimulation syndrome, investigation revealed large ovarian mass and increased tumor marker inhibin. Exploratory laparotomy was performed and revealed stage III ovarian cancer. The final pathology report indicated juvenile granulosa cell tumor. So, optimal surgical staging and cytoreductive surgery without fertility preserving were perfumed. Chemotherapy was recommended due to the advanced stage of ovarian cancer. Unfortunately, she experienced metastatic diseases in pelvic and abdomen in less than six months; and currently is receiving the second and third line chemotherapy.

**Conclusion:**

Persistent ovarian enlargement or ascites during or after infertility treatment should be carefully considered and managed.

## Introduction

The important side-effects of ovulation induction for infertility treatment included stimulation and multiple pregnancies; moreover, the possibility of increased mitotic activity of granulosa cells in the ovary must be notified (1). Granulosa cell tumors of the ovary are a rare entity among the neoplasms of gynecological oncology which arise from the sex-cord stromal cell of the ovary and the incidence rate of granulosa-cell tumors is different in various studies (2). The increased circulating concentration of estrogens is related to the ovulation stimulation by gonadotropins; perhaps it is contributable to the adverse effects. Unlike the epithelial ovarian cancer, the association of granulosa-cell tumors with increased gonadotropins has been reported in the studies (3). 

Accordingly, clomiphene citrate and human menopausal gonadotropins have been used since the early 1960s; it was expected that special ovarian tumors must be found in the coming decades than previously, but it really has not happened (4, 5). In one animal study, a correlation was found between gonadotropin exposures and coincidental granulosa-cell tumors (6). In another study, granulosa-cell tumor was reported in 12 patients after clomiphene’s ovarian induction; although the possibility of this special concern may be coincidental (7). 

The aim of this report is to report a case regarding the possible relation between anulosa-cell tumor and ovarian stimulation.

## Case report

**Figure 1:**
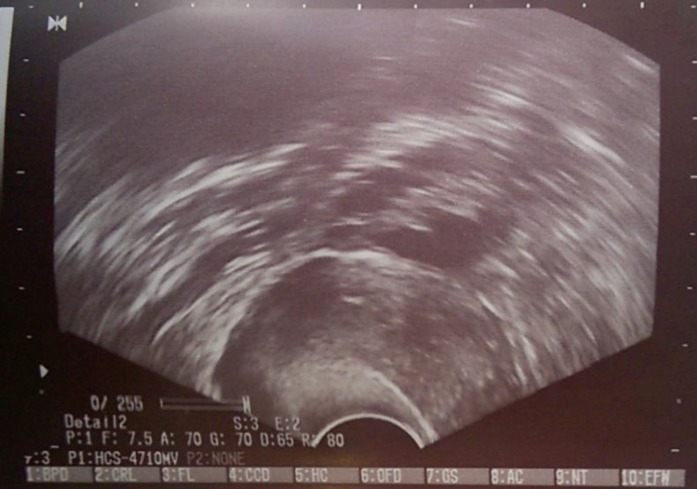
Sonography before induction of ovulation-normal ovary without dominant foliculs.

A 31 yr-old woman with complaints of massive abdominal distention and respiratory distress was referred to the gynecology and oncology department of an academic hospital, Mashhad University of Medical Sciences in Aug 2017. In past medical history, she mentioned a secondary infertility for four yrs and had one child aged eight yrs. The patient was candidate for In Vitro Fertilization (IVF) protocol due to tubal factors. In the first cycle of ovarian stimulation, metformin and Gonal-f 75 IU for six days were prescribed (Figure 1) and then continued for two days. The cycle was cancelled due to poor response after the second month from this protocol. She suffered from gradual abdominal distention. 

Despite the failure of IVF, she was under the outpatient care and supportive treatment with possible diagnosis of hyperstimulation syndrome. Therefore, antagonist GnRH was prescribed for two days. At the next delayed month visit, because of persistent symptoms with the probability of hyperthyroidism, she received gonadotropin hormone agonist (Decapeptyl). She was re-evaluated due to unresponsive to treatment within this period. 

**Figure 2:**
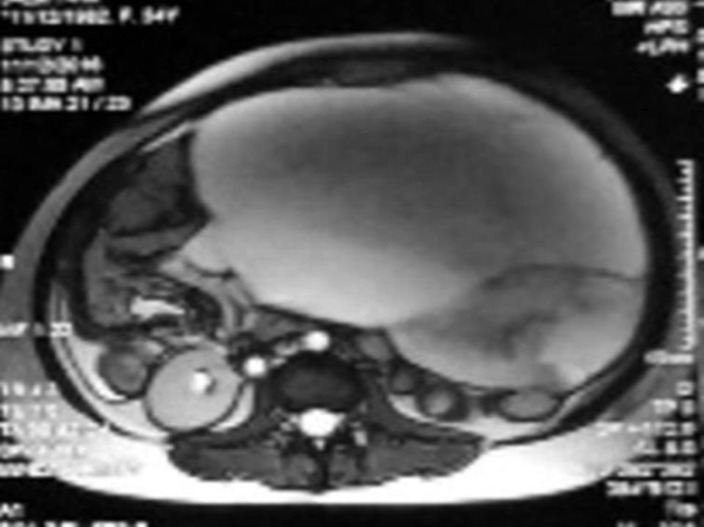
Multiple multiloculated cystic masses with predominantly solid components in both adnexa which were extended up to xiphoid.unfortunately I don’t have a better one.

Trans-abdominal and transvaginal ultrasonography were performed that showed multiple multiloculated cystic masses with predominantly solid components in both adnexa. The results of cross-sectional CT-scan and magnetic resonance imaging suggested the ovarian neoplasm. Also, massive peritoneal and pleural effusion was detected (Figure 2). In this time, 4 months after management of hyperstimulation syndrome, due to persistent large ovarian mass and increased tumor marker inhibin more than 3000 pg/mL, she was referred to our oncology department. Physical examination demonstrated enlarged masses extended up to hypogastric region which resembled 36 wks of pregnancy. 

**Figure 3:**
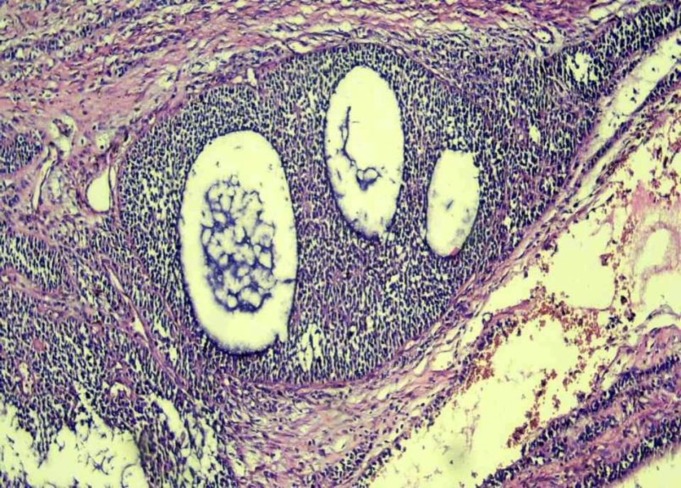
Histologic findings showed granulosa cell with ovoid nuclei and nuclear grooven with microfollicular pattern /HE*100MAGNIFIED 100 TIMES.

Exploratory laparotomy was performed that showed massive ascites fluid and multi solid cystic masses in both ovaries extended up to the Xiphoid. Complete resection of the tumor was done. Pathology report of frozen section was unable to confirm the malignancy. But, permanent histology indicated the tumor cells with round-to-ovoid nuclei and eosinophilic or vacuolated cytoplasm or microfollicular and trabecular. Moreover, Call-Exner bodies were observed in most areas which were compatible with juvenile granulosa cell tumors. A positive immunohistochemical staining for inhibin was the key point of this diagnostic feature (Figure 3). 

So, surgical staging surgery and optimal cytoreductive surgery without fertility preserving were done. At this stage, although pelvic and abdominal cavity appeared normal without any residual disease in the first surgery, one month later in the second surgery, we unpredictably encountered malignant tumor even in the omentum (stage IIIc of disease); so complete cytoreductive surgery was again performed. Then, three cycles of adjuvant chemotherapy with bleomycin, etoposide, and cisplatin (BEP) were prescribed. Unfortunately, she experienced metastatic diseases in pelvic and abdomen in less than six months, and currently is receiving the second line chemotherapy. Now the patient is under follow-up.

An informed consent was obtained from the patient for publication of this case report and accompanying images.

## Discussion

It is not clear that granulosa-cell tumor discovered during ovarian stimulation was a pre-existing tumor or incidentally caused by ovarian stimulation. Also, like as our case, it is unknown that tumor progression is due to drug effects or the natural phenomena of tumor led to cancer. Ovarian stimulation due to excess gonadotropins and its effects on the growth of ovarian surface may cause the carcinogenic effect. 

Since the gonadotropin receptors of both normal ovarian tissue and ovarian cancer are similar and also the tumor had a rapid progression, it is possible that superovulation affects the tumor growth (8). 

This phenomenon is one of the hypotheses of ovarian cancer in the early menopausal age, especially in the age group of 50-59 yr. Moreover, increased secretion of gonadotropins and development of cancer have been confirmed in animals (9). In addition, increased number of oocyte seems to be predisposed to the development of ovarian cancer. However, mechanism for this carcinogenic effect is unclear (10). Despite above mentioned, discovering of granulosa-cell tumors after ovarian stimulation might be caused due to hyperstimulation or truly random phenomenon. Moreover, considering the age of infertile patients, requesting for tumor markers, particularly germ cell group and sex cord tumors seems more logical. Also, increased marker inhibin-B is very diagnostic (11). The granulosa-cell tumors account for approximately 2-8% of all ovarian cancers (12). It seems that gynecologists who are specialist in infertility treatment were less likely to attend about the malignancy; they should be careful about the occurrence of persistent ovarian enlargement. 

Subsequently, exploratory laparotomy must be considered after radiological investigation and evaluation of tumor marker. After confirming the diagnosis of malignancy by frozen section, appropriate surgical staging and optimal cytoreductive surgery should be performed (13, 14). In the present case, unfortunately, frozen section could not help us and we had an obligation to perform the second surgery, and despite that pelvic and abdominal cavity appeared normal without any residual disease in the first surgery, but in the second surgery, we encountered malignant tumor even in the omentum (stage IIIc of disease); perhaps it was due to the tumor rapid nature of progression. Certainly, if malignancy was detected in the early stages, the need for adjuvant chemotherapy would be reduced (15). Diergaarde and colleagues in the evaluation of two recent large case-control studies in the United States did not find any contribution of ovarian cancer and fertility drug use (16). In literature review, there are conflicting results about the possible association between the use of infertility drugs and the increased risk of ovarian cancer. 

A relation between of granulosa-cell tumor and ovarian stimulation may have ruled out. In some studies reported that the possibility of overstimulation contributed to the development of tumor. Although we cannot prove a causal link between growth of ovarian tumor and ovarian stimulation Because our patient was not undergoing ovarian stimulation and ovarian tumors were identified during follow-up.

However, due to the potential additional risk associated with several reproductive factors influencing ovarian cancer risk, with regard to the different treatment schedules, the types and effects of certain drugs should be attended. Ideally, we emphasize on the strategies to improve the fertility rates without any risk of malignancy. In the current case, no relationship was found between ovarian stimulation and iatrogenic granulosa-cell tumor, but it is recommended that proper evaluation of patients before in vitro fertilization and careful monitoring of the ovaries during stimulation be considered as the standard of care in the infertility centers. In addition, if possible, post procedure surveillance and any suspected symptoms be assessed and long follow-up of these patients is suggested. In fact, the possibility of any relationship between fertility drugs and risk of ovarian cancer is an important question that requires further evaluation through large studies with sufficient follow-up time. Moreover, in the absence of pregnancy, any persistent and progressing enlargement of the ovary needs to be consulted with gynecologist oncologist at the first step and then, applying tumor markers and imaging modalities at the second step. 

## Conclusion

Persistent ovarian enlargement or ascites during or after infertility treatment should be carefully considered and managed.

## Conflict of interests

There is no conflict of interest in this regard.
